# Research on the relationship between common metabolic syndrome and meteorological factors in Wuhu, a subtropical humid city of China

**DOI:** 10.1186/s12889-023-17299-8

**Published:** 2023-11-29

**Authors:** Tao Zhang, Man Ni, Juan Jia, Yujie Deng, Xiaoya Sun, Xinqi Wang, Yuting Chen, Lanlan Fang, Hui Zhao, Shanshan Xu, Yubo Ma, Jiansheng Zhu, Faming Pan

**Affiliations:** 1 Department of Epidemiology and Biostatistics, School of Public Health, Anhui Medical University, 81 Meishan Road, Hefei, Anhui 230032 China; 2 The Key Laboratory of Major Autoimmune Diseases, Anhui Medical University, 81 Meishan Road, Hefei, Anhui 230032 China; 3 Department of Hospital Management Research, The First Affiliated Hospital of Anhui Medical University, Hefei, Anhui 230032 China; 4 Wuhu center for disease control and prevention, Wuhu, Anhui China; 5 Anhui Medical University, 81 Meishan Road, Hefei, Anhui 230032 China

**Keywords:** Metabolic syndrome, Climate variation, Short-term exposure effect

## Abstract

As climate conditions deteriorate, human health faces a broader range of threats. This study aimed to determine the risk of death from metabolic syndrome (MetS) due to meteorological factors. We collected daily data from 2014 to 2020 in Wuhu City, including meteorological factors, environmental pollutants and death data of common MetS (hypertension, hyperlipidemia and diabetes), as well as a total number of 15,272 MetS deaths. To examine the relationship between meteorological factors, air pollutants, and MetS mortality, we used a generalized additive model (GAM) combined with a distributed delay nonlinear model (DLNM) for time series analysis. The relationship between the above factors and death outcomes was preliminarily evaluated using Spearman analysis and structural equation modeling (SEM). As per out discovery, diurnal temperature range (DTR) and daily mean temperature (T mean) increased the MetS mortality risk notably. The ultra low DTR raised the MetS mortality risk upon the general people, with the highest RR value of 1.033 (95% CI: 1.002, 1.065) at lag day 14. In addition, T mean was also significantly associated with MetS death. The highest risk of ultra low and ultra high T mean occured on the same day (lag 14), RR values were 1.043 (95% CI: 1.010, 1.077) and 1.032 (95% CI: 1.003, 1.061) respectively. Stratified analysis’s result showed lower DTR had a more pronounced effect on women and the elderly, and ultra low and high T mean was a risk factor for MetS mortality in women and men. The elderly need to take extra note of temperature changes, and different levels of T mean will increase the risk of death. In warm seasons, ultra high RH and T mean can increase the mortality rate of MetS patients.

**Supplementary Information:**

The online version contains supplementary material available at 10.1186/s12889-023-17299-8.

## Introduction

Metabolic syndrome (MetS) is a cluster of common metabolic disorders, including high blood sugar, abnormal lipid levels, high blood pressure, and central obesity. These conditions are closely linked to increased risk of overall mortality and cardiovascular events. [1–3]. MetS affects more than 30% of adults worldwide [4]. However, discussing the key protection groups, the health burden of metabolic syndrome in the general population is also important. A review pointed out that the global prevalence of metabolic syndrome is estimated to be about one fourth of the world population. To put it differently,, over a billion people in the world are now affected by metabolic syndrome [5]. However, the risk of METS in specific populations cannot be ignored. Recent research indicates that MetS is emerging at an earlier age, affecting 3% of children and 5% of adolescents worldwide [6]. As a global health concern, MetS poses a substantial threat to public health and imposes a substantial economic burden [7]. Hence, it is imperative to investigate the risk factors contributing to the development of MetS and implement strategies for its prevention and control.

Meteorological factors are prevailing among recent researches. In the past few decades, carbon emissions have increased dramatically and continuously magnifying the greenhouse effect over the planet. As a result, global average temperatures continue to climb up which incurs a series of climate changes [8, 9]. This has also been accompanied by an increase in the rate and strength of extreme weather events (heat waves, droughts, floods and cold spells), which have seriously threatened the public health and, increased morbidity and mortality of various diseases [10]. Numerous studies have provided epidemiological evidence that the diurnal temperature range of single day exposure and continuous multi day cumulative exposure to extreme levels (extremely high or low) is positively correlated with the risk of all cause mortality, cardiovascular disease, and respiratory disease [11–14]. It is now generally accepted that environmental factors are important modulators of MetS. Studies have also discovered an increased risk of elevated fasting glucose and hypertriglyceridemia with prolonged exposure to higher ambient temperatures, potentially leading to the activation of metabolic mechanisms such as inflammation [15]. Another study in China also showed that ambient temperature can affect the relationship between air pollutants and MetS [16].

Same with China,as one of the countries with largest carbon emissons, the resulting climate and environmental issues have received much more attention now [17]. Diurnal temperature range (DTR), Mean temperature (T mean) and relative Humidity (RH) are important indicators to assess the state of meteorological change [18]. A review on the relationship between temperature and metabolic syndrome suggests that low ambient temperature may be an important risk factor for metabolic syndrome [19]. Another review from Mississippi shows an association between heat exposure and the prevalence of metabolic syndrome. Exposure to high temperatures reduces energy expenditure and may increase the prevalence of obesity and metabolic syndrome [20]. By reviewing published studies, most researches used meteorological factors as covariates, or only studied the relationship between a single meteorological factor and MetS prevalence or mortality. In order to fill the gap in this field, this study used the mortality data of Wuhu City from 2014 to 2020, included three meteorological factors (T mean, RH and DTR) for the first time, and used pollutant concentration as a covariate to explore the influence of meteorological elements on the mortality of three representative MetS (hypertension, hyperlipidemia and diabetes). The purpose of this study is to explore the association between meteorological factors and MetS mortality risk, and to provide a theoretical basis for formulating health and environmental governance policies, which could probably serve as a reference for related researches in this field.

## Materials and methods

### Basic information of the study cite

**Fig. 1:**
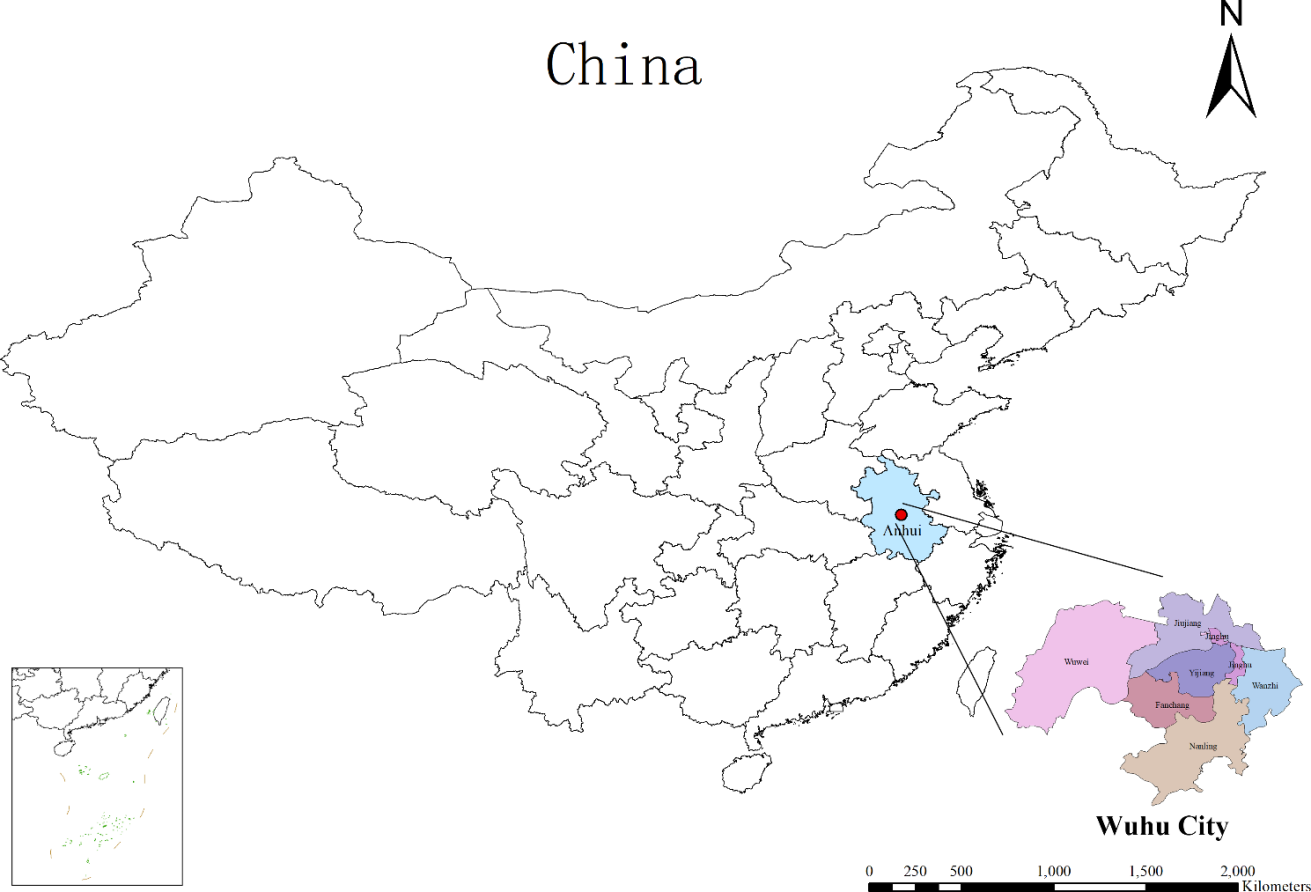
The geographical location and jurisdiction of Wuhu city

Wuhu City, located at the lower reaches of the Yangtze River, is engaged in China’s Yangtze River Delta urban agglomeration development planning for large cities. The landform is mainly dominated by terraces and plains, with abundant rainfall and four distinct seasons, belonging to a subtropical monsoon humid climate (Fig. 1). Data from the Wuhu Department of Statistics indicated that the city had a population of 3.644 million by 2020.

### Data collection and collation

The daily non-accidental mortality data from January 1, 2014, to December 31, 2020, were collected from the Wuhu Center for Disease Control and Prevention. The data were classified according to the International Statistical Classification of Diseases and Related Health Problems, tenth edition (ICD-10). Daily deaths due to hypertension (I10-I15), hyperlipidemia (E78), and diabetes (E10-E14) accounted for a total of 15,272 MetS-related deaths. Additionally, meteorological data and air pollutant data were collected from the Wuhu Meteorological Bureau and the environmental monitoring station, respectively (http://www.wuhu.gov.cn/public). Meteorological data is comprised of daily maximum temperature, minimum temperature, average temperature (T mean) and relative humidity (RH). DTR represents the temperature difference between the highest and lowest readings on the same day. The air pollutant data were selected from the average values of four local monitoring stations over the same time period, including particulate matter (PM_2.5_), inhalable particles (PM_10_), sulfur dioxide (SO_2_), nitrogen dioxide (NO_2_), ozone (O_3_) and carbon monoxide (CO).

### Statistical analysis method

**Fig. 2:**
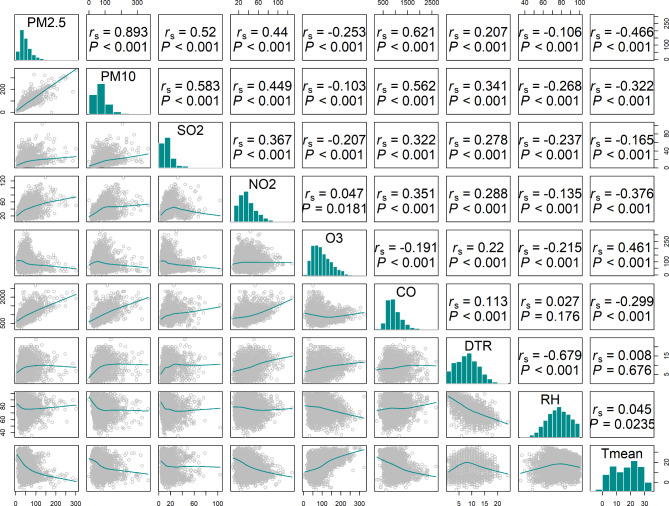
Spearman’s correlation coefficients meteorological factors and atmospheric pollutants: Spearman’s correlation coefficients at the top, distribution plot at the middle and scatter plot at the bottom

We collated the number of deaths incurred by major MetS (hypertension, hyperlipidemia, and diabetes), air pollutants (PM_2.5_, PM_10_, SO_2_, NO_2_, O_3_, and CO), and meteorological factors (DTR, T mean, and RH) for descriptive analysis. We used statistical measures including mean, standard deviation, maximum, minimum, median, and various percentiles (e.g., fifth percentile, twenty-fifth percentile, seventy-fifth percentile, ninety-fifth percentile) to describe the basic characteristics of each factor. It is essential to be cautious about collinearity when dealing with a large number of variables. Thus, We applied Spearman analysis to assess the correlations among these elements. When the coefficient of relation among the variables is more than 0.7, the correlation is perceived as a high level and the corresponding factors need to be excluded from building the model [21]. As it is shown on Fig. 2, PM_2.5_ and PM_10_ have a strong collinearity, which means only PM_2.5_ is included in the model for analysis. Structural equation model (SEM), though it is a linear model, is applied as a reference for the time series model to explore the relationship between death and other various factors [22]. The SEM model is formulated as follows:

$$\eta = \alpha + \Gamma {\text{X}} + \delta$$

In this formula, the number of MetS deaths is represented by $\eta$, while $\alpha$ is a constant term representing the intercept; ${\Gamma }$ demonstrates for the linear effect coefficient, and the latent predictive factor (DTR, T mean, RH, PM_2.5_, SO_2_, NO_2_, O_3_ and CO) is expressed by $\text{X}$. And the residual is represented by $\delta$.

Different climates may interact with each other, and the influence of climatic factors on human health is mostly nonlinear and complicated. The generalized additive model is flexible, and the daily death data often complies with the Poisson distribution, so we use DLNM to generate GAM to find the correlation between meteorological factors and common MetS deaths in China. The basic model is as below:

$${Y_t} \sim {\text{Poisson}}\left( {{\mu _t}} \right)$$

$$\begin{gathered} Log\left( {{\mu _t}} \right) = \alpha + \beta Tmea{n_{t,l}} + \gamma R{H_{t,l}} + \delta DT{R_{t,l}} + ns(Pollutan{t_t},df) \hfill \\+ ns(Time,df) + factor\left( {Holiday} \right) + factor\left( {DOW} \right) \hfill \\ \end{gathered}$$

Upon the above expression, ${\mu }_{t}$ represents the quantity of MetS death toll, *α* is the nodal increment of the formula, and *T mean*_*t,l*_, *RH*_*t,l*_ and *DTR*_*t,l*_ are used to represent the matrices generated in the DLNM model. While *t* and *l* are used to stand for the observation period and the lag days respectively. The *β*, *γ* and *δ* represent the vector coefficients of each matrix respectively, and the d-cubic spline function (CSF) is represented by *ns*(). The air pollutant concentration parameters are put together and represented by ${Pollutant}_{t}$ including particulate matter (PM_2.5_) and harmful gases (NO_2_, SO_2_, O_3_ and CO). The *df* stands for the degree of freedom, and the CSF controls the confounding effect of time and long-term trends, expressed by $ns(Time,df)$ [23]. At the same time, Holidays $factor\left(Holiday\right)$ and different days in a week $factor\left(DOW\right)$ are also controlled. We use Akaike’s information criterion (AIC) [24] to select the degree of freedom, and finally determine the annual degree of freedom is 7. According to previous studies on meteorological factors and MetS [25], a 14-day lag was selected based on the minimum AIC. Cumulative and one-day lag risk of death from MetS was expressed as RR and 95% CI. The meteorological factors were divided into four levels: ultra low (5th), low (25th), ultra high (95th), and high (75th).

The Wuhu City map was created using ArcMap 10.2 software. Data statistical analysis was performed using RGui (V4.1.2), and descriptive analysis was conducted using SPSS 23.0. The “DLNM” and “spline” packages were selected to match contaminant and meteorological factors models with time series. While the SEM is applied with the “lavaan” package of R software. The “Performance Analytics” package was applied to analyze the correlation between variables. Bilateral *p*-value < 0.05 was considered statistically significant.

## Results

### Baseline characteristics of data

Table 1 shows the number of deaths due to common metabolic syndrome (hypertension, hyperlipidemia and diabetes) among Wuhu City since 2014 to 2020 (2557 days totally), and the data features of climatic elements and major environmental contaminant. In the past few years, we have collected 15,272 death records on metabolic syndrome, with an average of 5.97 deaths per day. The sex ratio of death records was about 4:5 (male 6,975, 45.67%; female 8,297, 54.33%). With a boundary of 65 years old, the age ratio of the death toll was about 1:10 (0–65 years old 1,405, 9.20%; ≥ 65 years old 13,867, 90.8%). As a city located in the region of typical subtropical humid monsoon climate, the average absolute temperature difference of Wuhu is 8.66 ℃ (1 ℃ − 24 ℃), while the average temperature is 16.95 ℃ (-7 ℃ − 35 ℃), and the relative humidity is 76.68% (35.58 − 100%). The daily average concentrations of air pollutants were PM_2.5_: 50.35 µg/m^3^ (6.22–302 µg/m^3^); PM_10_: 74.98 µg/m^3^ (0–367.00 µg/m^3^); SO_2_: 15.48 µg/m^3^ (3.63–104.00 µg/m^3^); NO_2_: 37.93 µg/m^3^ (8–128.75 µg/m^3^); O_3_: 98.58 µg/m^3^ (6.94–324.47 µg/m^3^); CO: 957.60 µg/m^3^ (240.00–2670.00 µg/m^3^).

**Table 1:** Summary statistics of daily numbers of death, meteorological conditions and air pollutants in Wuhu.(2014 to 2020, 2557days)

Variables	Counts (%)	$\text{M}\text{e}\text{a}\text{n} \pm \text{S}\text{D}$	Centiles						
			Minimum	*P*_5_	*P*_25_	Median	*P*_75_	*P*_95_	Maximum
**Metabolic syndrome**									
Total	15,272 (100.00)	5.97 ± 3.29	0	1	4	6	8	12	27
Male	6975 (45.67)	2.73 ± 1.92	0	0	1	2	4	6	12
Female	8297 (54.33)	3.24 ± 2.20	0	0	2	3	5	7	16
0–65 years	1405 (9.20)	0.55 ± 0.78	0	0	0	0	1	2	8
≥ 65 years	13,867 (90.8)	5.42 ± 3.08	0	1	3	5	7	11	22
**Meteorological conditions**									
DTR (℃)	-	8.66 ± 4.23	1.00	2.00	5.00	9.00	12.00	16.00	24.00
T mean (℃)	-	16.95 ± 9.09	-7.00	2.50	8.96	17.50	24.50	31.00	35.00
RH (%)	-	76.68 ± 12.32	35.38	55.96	67.96	76.88	86.00	96.00	100.00
**Air pollutants**									
PM_2.5_ (µg/m^3^)	1055 (41.26)	50.35 ± 32.46	6.22	16.12	28.10	42.11	63.58	114.29	302.00
PM_10_ (µg/m^3^)	306 (11.97)	74.98 ± 41.88	0.00	28.00	45.67	65.35	94.24	153.58	367.00
SO_2_ (µg/m^3^)	6 (0.23)	15.48 ± 10.30	3.63	5.37	8.33	13.00	18.88	37.19	104.00
NO_2_ (µg/m^3^)	280 (10.95)	37.93 ± 17.14	8.00	16.03	25.15	34.99	47.38	70.70	128.75
O_3_ (µg/m^3^)	909 (35.55)	98.58 ± 50.35	6.94	33.00	59.38	88.57	129.90	196.68	324.47
CO (µg/m^3^)	1 (0.04)	957.60 ± 311.89	240.00	570.00	740.00	900.00	1120.00	1550.00	2670.00

Abbreviations: SD: standard deviation; DTR: diurnal temperature range; RH: relative humidity; T mean: temperature mean; PM_2.5_: particulate matter ≤ 2.5 μm in aerodynamic diameter; PM_10_: particulate matter ≤ 10 μm in aerodynamic diameter; SO_2_: sulfur dioxide; NO_2_: nitrogen dioxide; CO: carbon monoxide; O_3_: ozone; Counts of Air pollutants: number and proportion of days with each air pollutant as a daily major air pollutant

In order to explore the daily main pollutants in the region and their proportion in the whole year, the air quality index (AQI) for every contaminant was determined. PM_2.5_ is the most prominent air pollutant, accounting for 41.26% of the main pollutant days. The proportions of O_3_, PM_10_ and NO_2_ were 35.55%, 11.97% and 10.95% respectively. SO_2_ and CO had the lowest occurrence among all the main pollutants, 6 days and 1 day respectively.

### Correlation analysis and structural equation model

**Fig. 3:**
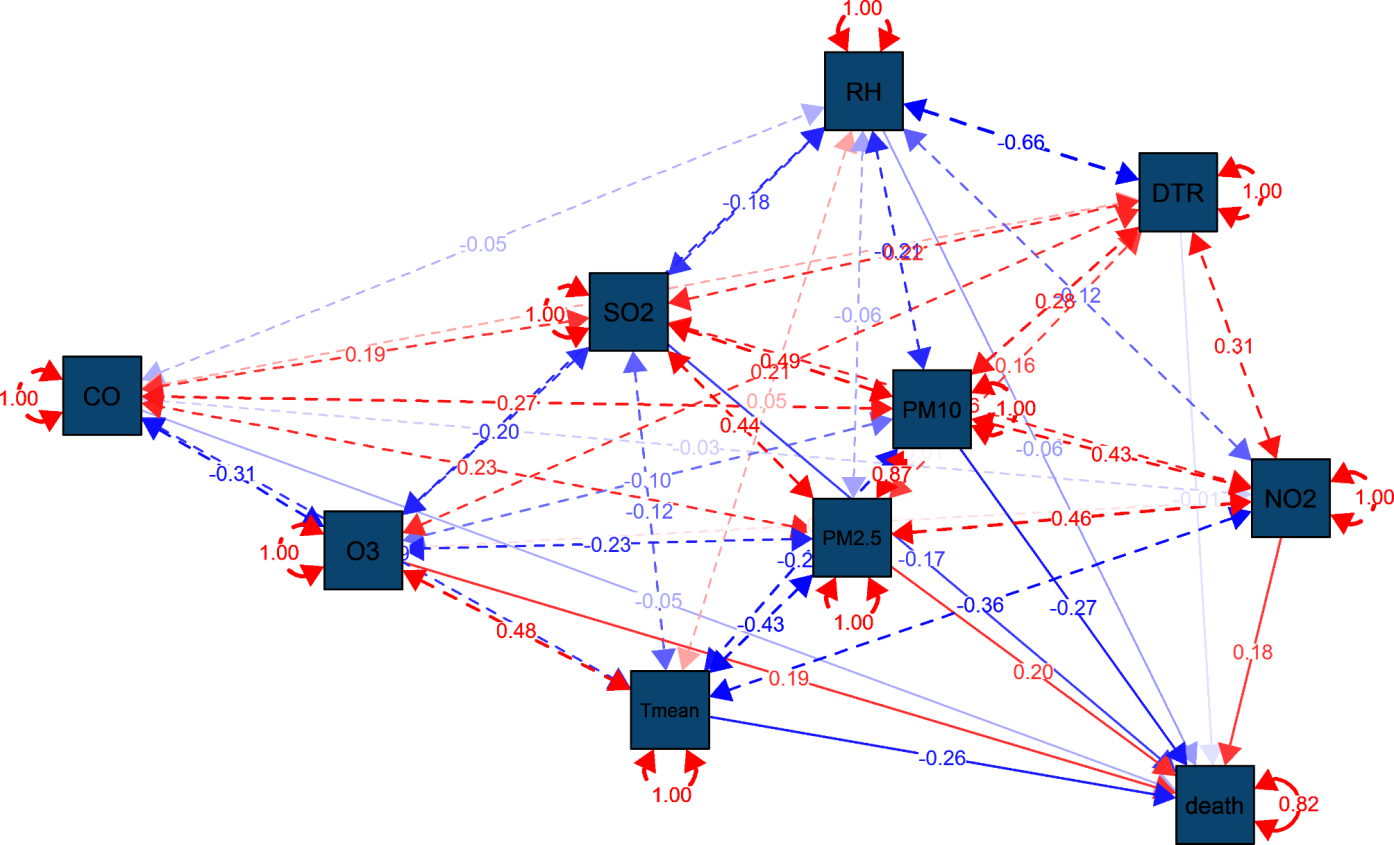
SEM analysis of the direct and indirect climate effects on metabolic syndrome mortality

Figure 2 expresses the results of Spearman analysis among air contaminants and meteorological elements. DTR had a positive correlation with NO_2_, O_3_ and CO (*P* < 0.001). And T mean was positively correlated with O_3_, and negatively correlated with PM_2.5_, PM_10_, and NO_2_ (*P* < 0.001). However, there was no significant correlation between RH and CO, and it was negatively correlated with O_3_ (*P* < 0.001). Among the air pollutants included in the study, PM_2.5_ was positively correlated with PM_10_ (*r*_*s*_ > 0.7, *P* < 0.001), and had a negative correlation with O_3_ and T mean. In addition, there exists a significant negative relation between DTR and RH. The results of SEM to explore the relationship between MetS mortality risk and meteorological factors and air pollutants are shown in Fig. 3. Among all the factors, NO_2_, O_3_ and PM_2.5_ had positive effects, while PM_10_, T mean, SO_2_, RH, CO and DTR had negative effects. And the absolute value of the standardized loading factor (SLC) was larger in PM_10_ (0.27), T mean (0.26), PM_2.5_ (0.20), O_3_ (0.19), NO_2_ (0.18) and SO_2_ (0.17).

There is a strong correlation between the above factors and the risk of MetS death. However, SEM has insufficient ability to capture nonlinear relationships, of which the results should be taken cautiously.

### Effects of meteorological factors on mortality risk of common metabolic syndrome

**Fig. 4:**
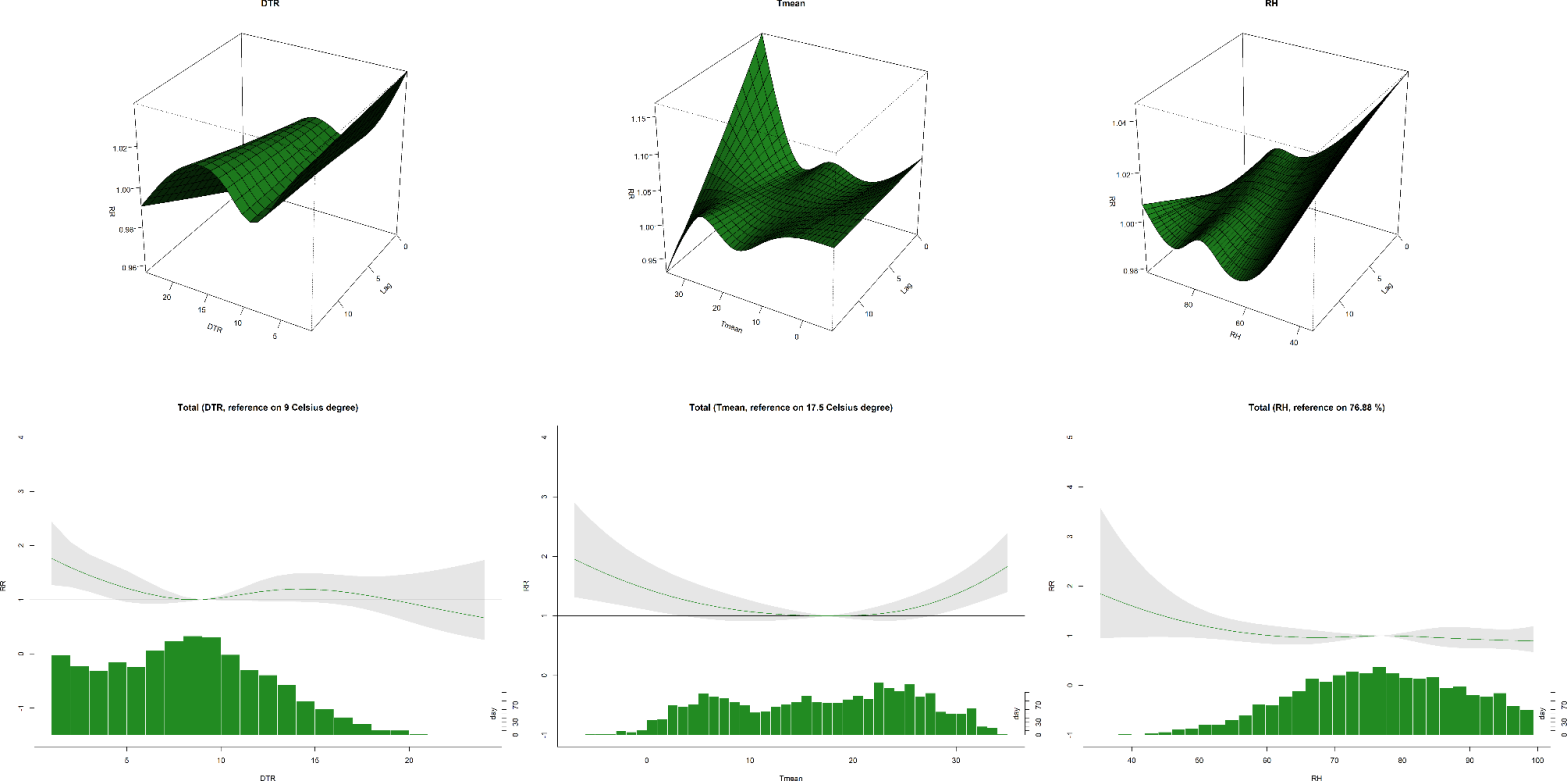
The 3D graph, and overall exposure-response association curve between DTR, RH, T mean and metabolic syndrome mortality

Figure 4 shows the association between the risk of death of common MetS and DTR, T mean and RH. In this study, DTR and T mean had a positive relationship with the metabolic syndrome death risk, while no pronounced relation was found between RH and the death risk of metabolic syndrome. The results are shown in Table 2. Taking the 50th percentile (9 ℃) of DTR as the reference value among the single-day lag effect model, the impact of the ultra low value (5th percentile) of DTR exists throughout the day 1 to day 14 with an increasing trend. The highest RR value of DTR occured at the lag day 14 (RR: 1.033, 95% CI: 1.002, 1.065). As for cumulative lag effect modeling, ultra low value (5th percentile) of DTR was found to be significantly correlated with the death risk of MetS (*P* < 0.05), and the value of RR gradually increased to reach the highest value 1.585 (lag 0–14, 95% CI: 1.226, 2.049) during the short-term time. Except for ultra low DTR, no significant association between other levels of DTR and MetS death risk was found.

**Table 2:** Relative risk (RR) of Common metabolic syndrome daily death for specific DTR on different lag days

	single-day lag						cumulative-day lag			
lag	5th percentile	25th percentile	75th percentile	95th percentile		lag	5th percentile	25th percentile	75th percentile	95th percentile
0	1.029 (0.998, 1.061)	1.009 (0.980, 1.038)	1.003 (0.991, 1.016)	1.000 (0.973, 1.028)		0–0	1.029 (0.998, 1.061)	1.009 (0.980, 1.038)	1.003 (0.991, 1.016)	1.000 (0.973, 1.028)
1	1.030 (1.001, 1.058)*	1.009 (0.984, 1.035)	1.004 (0.993, 1.015)	1.001 (0.977, 1.027)		0–1	1.060 (1.000, 1.123)	1.018 (0.964, 1.074)	1.007 (0.984, 1.030)	1.001 (0.950, 1.055)
2	1.030 (1.005, 1.056)*	1.010 (0.987, 1.033)	1.004 (0.994, 1.014)	1.003 (0.980, 1.026)		0–2	1.091 (1.005, 1.185)*	1.028 (0.951, 1.110)	1.011 (0.978, 1.045)	1.004 (0.932, 1.082)
3	1.030 (1.007, 1.053)*	1.010 (0.990, 1.032)	1.005 (0.995, 1.013)	1.004 (0.984, 1.025)		0–3	1.124 (1.012, 1.248)*	1.038 (0.942, 1.145)	1.015 (0.974, 1.058)	1.008 (0.917, 1.108)
4	1.030 (1.010, 1.051)*	1.011 (0.992, 1.030)	1.005 (0.996, 1.013)	1.006 (0.987, 1.024)		0–4	1.158 (1.023, 1.311)*	1.050 (0.935, 1.179)	1.020 (0.971, 1.071)	1.014 (0.906, 1.134)
5	1.031 (1.012, 1.050)*	1.011 (0.994, 1.029)	1.005 (0.997, 1.012)	1.007 (0.990, 1.024)		0–5	1.194 (1.037, 1.375)*	1.062 (0.931, 1.212)	1.025 (0.969, 1.084)	1.021 (0.898, 1.160)
6	1.031 (1.013, 1.049)*	1.012 (0.996, 1.029)	1.005 (0.998, 1.012)	1.008 (0.993, 1.024)		0–6	1.231 (1.053, 1.439)*	1.075 (0.929, 1.244)	1.030 (0.968, 1.096)	1.030 (0.894, 1.186)
7	1.031 (1.014, 1.049)*	1.013 (0.997, 1.029)	1.005 (0.999, 1.012)	1.010 (0.994, 1.025)		0–7	1.269 (1.071, 1.504)*	1.089 (0.929, 1.275)	1.035 (0.968, 1.108)	1.040 (0.892, 1.212)
8	1.031 (1.014, 1.050)*	1.013 (0.997, 1.030)	1.006 (0.999, 1.013)	1.011 (0.996, 1.027)		0–8	1.309 (1.091, 1.570)*	1.103 (0.931, 1.307)	1.041 (0.969, 1.119)	1.051 (0.892, 1.239)
9	1.032 (1.013, 1.051)*	1.014 (0.997, 1.031)	1.006 (0.999, 1.013)	1.013 (0.996, 1.030)		0–9	1.350 (1.113, 1.638)*	1.119 (0.935, 1.339)	1.047 (0.970, 1.131)	1.065 (0.894, 1.267)
10	1.032 (1.011, 1.053)*	1.015 (0.996, 1.033)	1.006 (0.998, 1.014)	1.014 (0.996, 1.033)		0–10	1.394 (1.137, 1.709)*	1.135 (0.939, 1.372)	1.054 (0.972, 1.143)	1.080 (0.898, 1.297)
11	1.032 (1.009, 1.056)*	1.015 (0.995, 1.036)	1.006 (0.998, 1.015)	1.016 (0.995, 1.036)		0–11	1.439 (1.160, 1.784)*	1.152 (0.944, 1.407)	1.060 (0.974, 1.155)	1.096 (0.904, 1.330)
12	1.033 (1.007, 1.059)*	1.016 (0.993, 1.039)	1.007 (0.997, 1.016)	1.017 (0.995, 1.040)		0–12	1.485 (1.184, 1.864)*	1.171 (0.949, 1.444)	1.068 (0.976, 1.168)	1.115 (0.909, 1.367)
13	1.033 (1.005, 1.062)*	1.016 (0.991, 1.042)	1.007 (0.996, 1.018)	1.018 (0.994, 1.044)		0–13	1.534 (1.206, 1.952)*	1.190 (0.953, 1.485)	1.075 (0.977, 1.182)	1.135 (0.915, 1.409)
14	1.033 (1.002, 1.065)*	1.017 (0.989, 1.046)	1.007 (0.995, 1.019)	1.020 (0.992, 1.048)		0–14	1.585 (1.226, 2.049)*	1.210 (0.956, 1.532)	1.083 (0.979, 1.198)	1.158 (0.920, 1.457)

The table records use the mean of RR values and 95% confidence intervals; *P < 0.05

Taking the median of T mean (17.5 ℃) for a reference, Table 3 shows the results that the single-day lag influences of ultra low and high values of T mean continued to exist and increase for 7 days and 6 days respectively (*P* < 0.05). The highest risk occured on the lag day 14, RR values were 1.043 (95% CI: 1.010, 1.077) and 1.032 (95% CI: 1.003, 1.061) respectively. The ultra high T mean was positively correlated with MetS, which lasted for 9 days and decreased day by day. The highest impact of T mean occured at the beginning with the RR value of 1.070 (lag 0, 95% CI: 1.027, 1.115). Low T mean (25th percentile) was not found to significantly affect the risk of MetS death in the one-day lag model. The cumulative lag model showed that ultra low T mean had a positive association with the MetS death risk, with a maximum RR of 1.291 (lag 0–14, 95% CI: 1.004, 1.659). The ultra high T mean significantly raised the danger of MetS death, starting since lag 0–0 to lag 0–14, and the maximum RR value was 1.531 (lag 0–11, 95% CI: 1.248, 1.878).

**Table 3:** Relative risk (RR) of Common metabolic syndrome daily death for specific T mean on different lag days

	single-day lag						cumulative-day lag			
lag	5th percentile	25th percentile	75th percentile	95th percentile		lag	5th percentile	25th percentile	75th percentile	95th percentile
0	0.992 (0.956, 1.030)	0.986 (0.960, 1.012)	0.983 (0.955, 1.011)	1.070 (1.027, 1.115)*		0–0	0.992 (0.956, 1.030)	0.986 (0.960, 1.012)	0.983 (0.955, 1.011)	1.070 (1.027, 1.115)*
1	0.996 (0.963, 1.029)	0.989 (0.966, 1.012)	0.986 (0.961, 1.011)	1.064 (1.026, 1.103)*		0–1	0.988 (0.921, 1.060)	0.975 (0.928, 1.025)	0.969 (0.918, 1.022)	1.139 (1.054, 1.231)*
2	0.999 (0.970, 1.029)	0.992 (0.971, 1.012)	0.989 (0.968, 1.011)	1.058 (1.025, 1.091)*		0–2	0.988 (0.894, 1.091)	0.967 (0.901, 1.038)	0.958 (0.889, 1.034)	1.205 (1.081, 1.343)*
3	1.003 (0.977, 1.029)	0.994 (0.976, 1.013)	0.993 (0.975, 1.012)	1.052 (1.024, 1.080)*		0–3	0.990 (0.874, 1.122)	0.961 (0.880, 1.050)	0.952 (0.866, 1.045)	1.267 (1.107, 1.450)*
4	1.006 (0.984, 1.030)	0.997 (0.981, 1.013)	0.996 (0.981, 1.012)	1.045 (1.022, 1.069)*		0–4	0.997 (0.861, 1.155)	0.958 (0.864, 1.064)	0.948 (0.850, 1.057)	1.324 (1.132, 1.549)*
5	1.010 (0.990, 1.030)	0.997 (0.986, 1.014)	1.000 (0.987, 1.013)	1.039 (1.020, 1.059)*		0–5	1.007 (0.853, 1.188)	0.958 (0.852, 1.077)	0.948 (0.840, 1.070)	1.376 (1.157, 1.637)*
6	1.014 (0.996, 1.032)	1.002 (0.990, 1.015)	1.003 (0.992, 1.015)	1.033 (1.017, 1.050)*		0–6	1.020 (0.851, 1.224)	0.960 (0.845, 1.092)	0.951 (0.835, 1.084)	1.422 (1.180, 1.713)*
7	1.017 (1.000, 1.034)	1.005 (0.993, 1.017)	1.007 (0.996, 1.018)	1.027 (1.012, 1.042)*		0–7	1.038 (0.854, 1.261)	0.965 (0.842, 1.107)	0.958 (0.835, 1.099)	1.460 (1.201, 1.775)*
8	1.021 (1.004, 1.038)*	1.008 (0.996, 1.020)	1.010 (0.999, 1.022)	1.021 (1.005, 1.037)*		0–8	1.059 (0.863, 1.300)	0.973 (0.843, 1.124)	0.968 (0.840, 1.115)	1.491 (1.220, 1.822)*
9	1.024 (1.007, 1.043)*	1.011 (0.997, 1.024)	1.014 (1.001, 1.027)*	1.015 (0.997, 1.033)		0–9	1.085 (0.877, 1.343)	0.983 (0.847, 1.142)	0.981 (0.850, 1.133)	1.513 (1.235, 1.854)*
10	1.028 (1.008, 1.048)*	1.013 (0.998, 1.029)	1.018 (1.002, 1.033)*	1.009 (0.987, 1.031)		0–10	1.116 (0.895, 1.391)	0.997 (0.854, 1.164)	0.999 (0.863, 1.155)	1.526 (1.245, 1.872)*
11	1.032 (1.009, 1.055)*	1.016 (0.999, 1.034)	1.021 (1.003, 1.040)*	1.003 (0.977, 1.029)		0–11	1.151 (0.917, 1.444)	1.013 (0.863, 1.188)	1.020 (0.881, 1.181)	1.531 (1.248, 1.878)*
12	1.035 (1.010, 1.062)*	1.019 (0.999, 1.039)	1.025 (1.003, 1.047)*	0.997 (0.967, 1.028)		0–12	1.191 (0.943, 1.505)	1.032 (0.875, 1.218)	1.045 (0.901, 1.212)	1.526 (1.242, 1.875)*
13	1.039 (1.010, 1.069)*	1.022 (0.999, 1.045)	1.028 (1.003, 1.054)*	0.991 (0.957, 1.027)		0–13	1.238 (0.973, 1.575)	1.055 (0.888, 1.253)	1.074 (0.922, 1.252)	1.513 (1.226, 1.868)*
14	1.043 (1.010, 1.077)*	1.025 (0.999, 1.050)	1.032 (1.003, 1.061)*	0.985 (0.946, 1.026)		0–14	1.291 (1.004, 1.659)*	1.081 (0.901, 1.295)	1.109 (0.944, 1.302)	1.491 (1.196, 1.859)*

The table records use the mean of RR values and 95% confidence intervals; *P < 0.05

As shown in Table 4, we failed to observe a statistically significant association between RH and MetS in the study. However, as shown in Fig. 4, there exists an uptrend on the left side among the risk curved line of RH. Thus, from the overall population, the likelihood of Mets mortality incurred by exposure to lower RH cannot be ruled out.

**Table 4:** Relative risk (RR) of Common metabolic syndrome daily death for specific RH on different lag days

	single-day lag						cumulative-day lag			
lag	5th percentile	25th percentile	75th percentile	95th percentile		lag	5th percentile	25th percentile	75th percentile	95th percentile
0	1.018 (0.994, 1.043)	1.005 (0.989, 1.022)	1.002 (0.979, 1.025)	0.987 (0.960, 1.016)		0–0	1.018 (0.994, 1.043)	1.005 (0.989, 1.022)	1.002 (0.979, 1.025)	0.987 (0.960, 1.016)
1	1.016 (0.994, 1.039)	1.004 (0.990, 1.019)	1.001 (0.980, 1.022)	0.988 (0.964, 1.014)		0–1	1.035 (0.988, 1.083)	1.009 (0.979, 1.041)	1.003 (0.959, 1.048)	0.976 (0.925, 1.030)
2	1.015 (0.995, 1.035)	1.003 (0.990, 1.016)	1.001 (0.982, 1.020)	0.989 (0.967, 1.012)		0–2	1.050 (0.984, 1.121)	1.013 (0.969, 1.058)	1.003 (0.942, 1.068)	0.966 (0.895, 1.042)
3	1.013 (0.995, 1.031)	1.002 (0.991, 1.014)	1.000 (0.983, 1.017)	0.990 (0.971, 1.011)		0–3	1.063 (0.979, 1.155)	1.015 (0.960, 1.073)	1.003 (0.926, 1.087)	0.957 (0.869, 1.053)
4	1.011 (0.995, 1.027)	1.001 (0.991, 1.012)	0.999 (0.984, 1.015)	0.991 (0.974, 1.010)		0–4	1.075 (0.975, 1.186)	1.016 (0.952, 1.085)	1.003 (0.912, 1.103)	0.948 (0.847, 1.062)
5	1.009 (0.995, 1.024)	1.000 (0.991, 1.010)	0.999 (0.985, 1.014)	0.992 (0.976, 1.009)		0–5	1.085 (0.972, 1.213)	1.017 (0.944, 1.095)	1.002 (0.899, 1.116)	0.941 (0.828, 1.071)
6	1.008 (0.995, 1.021)	0.999 (0.990, 1.008)	0.998 (0.985, 1.012)	0.993 (0.978, 1.009)		0–6	1.094 (0.968, 1.236)	1.016 (0.936, 1.102)	1.000 (0.886, 1.128)	0.935 (0.811, 1.078)
7	1.006 (0.993, 1.019)	0.998 (0.990, 1.007)	0.998 (0.985, 1.011)	0.994 (0.980, 1.009)		0–7	1.100 (0.964, 1.256)	1.014 (0.928, 1.108)	0.998 (0.875, 1.138)	0.930 (0.798, 1.084)
8	1.004 (0.991, 1.017)	0.997 (0.989, 1.006)	0.997 (0.984, 1.011)	0.995 (0.981, 1.011)		0–8	1.105 (0.960, 1.272)	1.012 (0.921, 1.112)	0.995 (0.864, 1.146)	0.926 (0.786, 1.090)
9	1.003 (0.989, 1.017)	0.996 (0.987, 1.006)	0.997 (0.983, 1.011)	0.996 (0.981, 1.013)		0–9	1.108 (0.955, 1.285)	1.008 (0.912, 1.113)	0.992 (0.854, 1.154)	0.922 (0.776, 1.096)
10	1.001 (0.986, 1.016)	0.995 (0.985, 1.006)	0.996 (0.981, 1.012)	0.997 (0.980, 1.015)		0–10	1.109 (0.949, 1.296)	1.003 (0.904, 1.114)	0.989 (0.843, 1.160)	0.920 (0.768, 1.102)
11	0.999 (0.982, 1.016)	0.994 (0.983, 1.006)	0.996 (0.979, 1.013)	0.998 (0.979, 1.018)		0–11	1.108 (0.941, 1.304)	0.998 (0.894, 1.113)	0.985 (0.832, 1.166)	0.919 (0.760, 1.110)
12	0.997 (0.979, 1.016)	0.993 (0.981, 1.006)	0.995 (0.977, 1.014)	0.999 (0.978, 1.022)		0–12	1.105 (0.931, 1.312)	0.991 (0.883, 1.112)	0.980 (0.820, 1.171)	0.918 (0.753, 1.120)
13	0.996 (0.975, 1.017)	0.993 (0.978, 1.007)	0.995 (0.974, 1.016)	1.000 (0.976, 1.025)		0–13	1.100 (0.918, 1.318)	0.984 (0.871, 1.111)	0.975 (0.807, 1.178)	0.919 (0.745, 1.133)
14	0.994 (0.971, 1.018)	0.992 (0.976, 1.007)	0.994 (0.972, 1.017)	1.001 (0.974, 1.029)		0–14	1.094 (0.903, 1.325)	0.975 (0.857, 1.110)	0.970 (0.793, 1.186)	0.920 (0.737, 1.149)

The table records use the mean of RR values and 95% confidence intervals; *P < 0.05

### Gender, age and season subgroup analysis

**Fig. 5:**
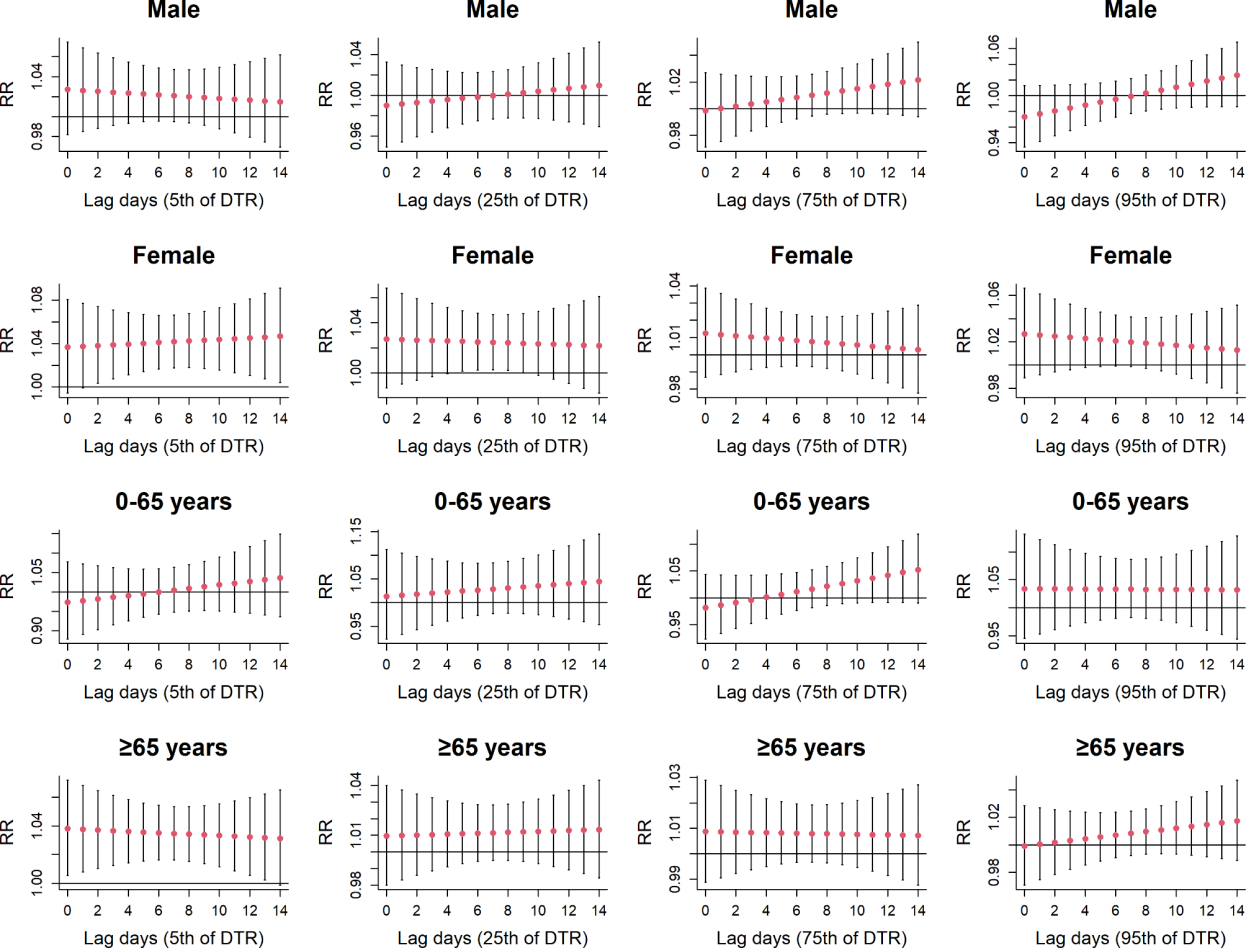
The lagged effects of DTR on metabolic syndrome mortality at various lag days

The hierarchical analysis’ results of DTR using gender and age are shown in Fig. 5. We found that DTR had a significant relationship with the MetS death risk in women and the elderly, but not in men and young people. As for women exposed to ultra low DTR, the MetS death risk increased gradually reaching a peak at a lag of 14 days (RR: 1.047, 95% CI: 1.004, 1.091). However, when exposed in low DTR environment, the danger of MetS death in female increased and expressed a down trend, and the maximum RR value appeared at the lag day 5 (RR: 1.025, 95% CI: 1.001, 1.049). Compared with young people, ultra low DTR was more likely to increase the risk of MetS death regarding with the elderly people, with the greatest risk occurring at the beginning of this short-term (lag 0, RR: 1.035, 95% CI: 1.003, 1.069). The results of stratified analysis based on the hot and cold seasons are shown in the supplementary materials. During the cold season, ultra low DTR are risk factors for death in MetS patients. (Supplementary Fig. 1). During the warm season, no significant statistical correlation was found between different levels of DTR and the overall population MetS mortality rate (Supplementary Fig. 2).

**Fig. 6:**
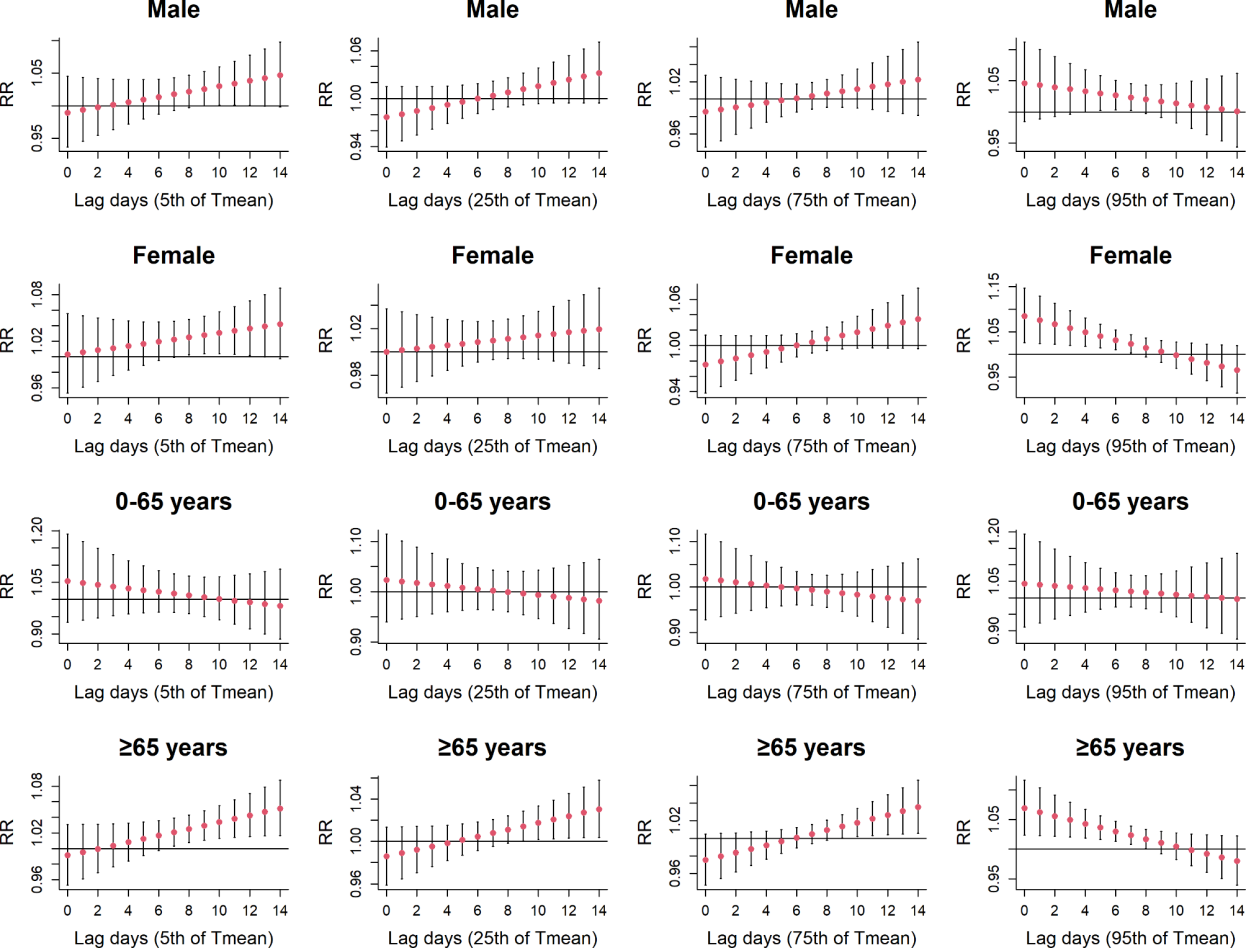
The lagged effects of T mean on metabolic syndrome mortality at various lag days

Figure 6 shows the existence of correlation between T mean and MetS mortality risk according to different genders and ages. In addition to young people, we found that T mean could affect men, women and elderly significantly. For men, both ultra low and high T mean exposures increased the risk of MetS death, with the highest risk of death taking place at lag day 11 (RR: 1.033, 95% CI: 1.000, 1.068) and lag day 4 (RR: 1.034, 95% CI: 1.001, 1.069) respectively. A similar association was also observed in women. When exposed to ultra low T mean, women’s risk of death from MetS increased with lag days with a greatest RR value of 1.039 (95% CI: 1.000, 1.080, lag 13). When influenced by ultra high T mean, the highest death risk in female MetS happened on the first day among the short period (RR: 1.089, 95% CI: 1.030, 1.151). Regarding the elderly, different levels of T mean were significantly associated with the risk of MetS death. At ultra low, low and high T mean, the MetS death risk increased with the lag days, and the greatest RR values were 1.052 (95% CI: 1.017, 1.088, lag 14), 1.032 (95% CI: 1.005, 1.059, lag 14) and 1.038 (95% CI: 1.008, 1.069,lag 14) respectively. When exposed to ultra high T mean, the danger of MetS showed a decreasing trend, with a greatest RR value of 1.074 (lag 0, 95% CI: 1.029, 1.121). The results of Supplementary Fig. 1 show that there is no statistically significant correlation between ultra low T mean and the risk of death in MetS patients during the cold season, while lower T mean increase MetS mortality. In the warm season, lower daily average temperatures show a protective effect on MetS patients, while ultra high T mean increase the mortality rate of MetS patients (Supplementary Fig. 2).

**Fig. 7:**
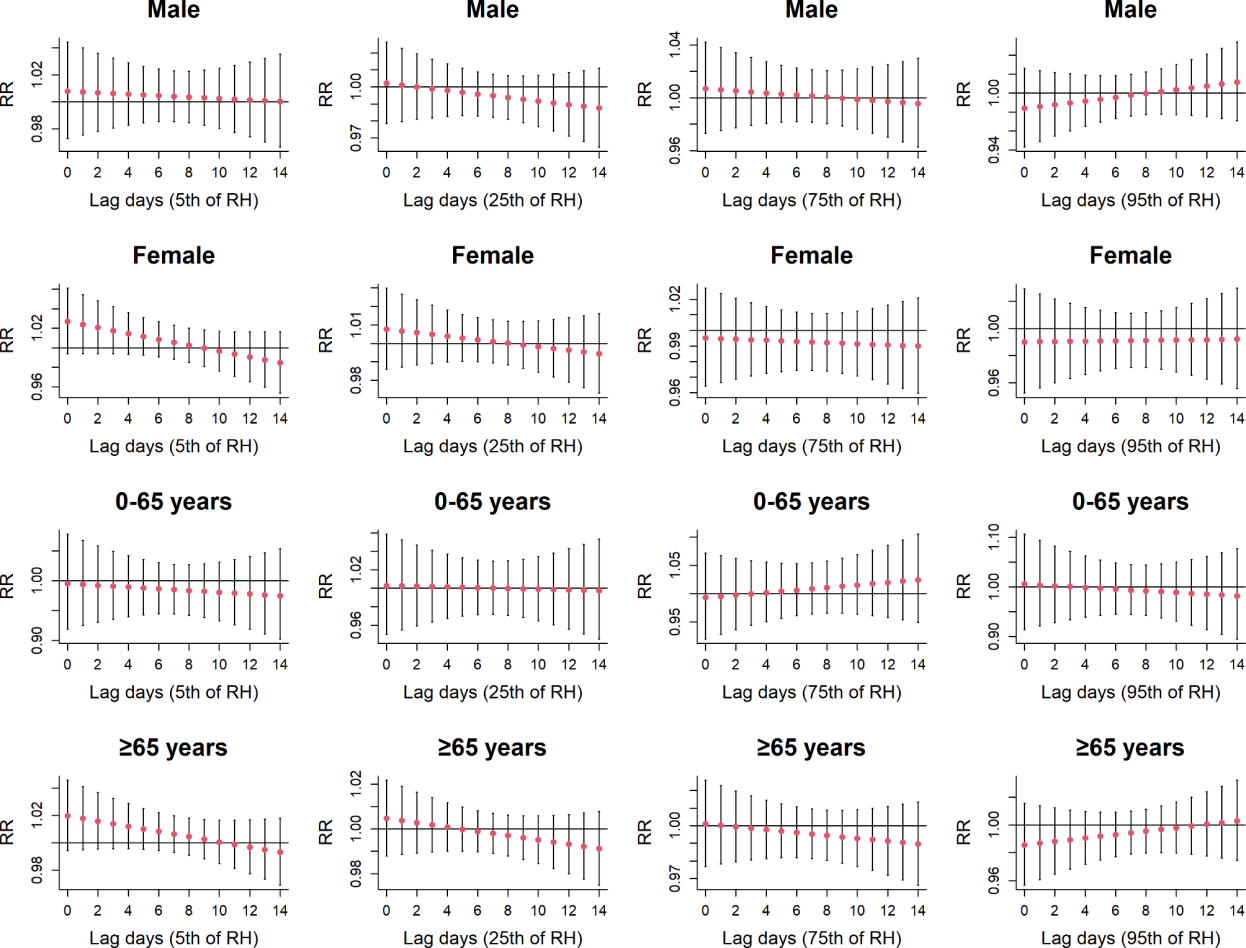
The lagged effects of RH on metabolic syndrome mortality at various lag days

The results of the stratified analysis regarding the association between relative humidity (RH) and MetS are displayed in Fig. 7. The figure does not reveal any significant associations between different RH levels and various populations. However, it’s noteworthy that in comparison to ultra high RH, the relative risk (RR) associated with MetS-related mortality significantly increases in women and the elderly exposed to ultra low RH. Thus, we cannot rule out the possibility that extreme RH may elevate the risk of death. Supplementary Fig. 1 shows no significant correlation between different RH levels and mortality in MetS patients during cold seasons. In contrast, in warm seasons, exposure to ultra high RH raises the risk of death in MetS patients, as indicated in Supplementary Fig. 2.

## Discussion

In recent years, with the rapid development of the Yangtze River Delta Economic Belt, the population has continued to grow. This has led to the intensification of the urban heat island effect and an increase in extreme weather events Severe air pollution is the main health risk problem we are currently faced with. The influence of climatic variation on human health has also received much more attention now. After adjusting the effects of air pollutants in this research, we first studied whether there was a significant correlation between DTR, T mean, RH and MetS death risk in Wuhu City. The association analysis results of air pollutants and meteorological factors are displayed in Fig. 2, DTR is negatively related to RH. Although the specific mechanism of the interaction of meteorological factors still remains not clear [26], we speculate that it is associated with the geographical situation and climatic characteristics of Wuhu City. More importantly, the average daily temperature difference in summer of Wuhu is small, and the RH is higher when DTR is low according to records from monitoring sites. [27]. The overall results of Fig. 4 showed that short-term exposure to ultra low DTR raised the MetS death risk, while exposure to ultra low, high and ultra high T mean also had a association with the increased danger of MetS death significantly.

We examined the relationship between MetS death risk and DTR, presenting the outcomes in. Table 2. Notably, ultra low DTR increased MetS death risk, while no significant correlation was observed at other DTR levels. In Table 2, it’s evident that under the influence of ultra low DTR, the risk RR value for cumulative lag effect significantly surpasses that of single-day lag effect. A study on MetS in southwest China found a mortality increase in hypertensive patients associated with high DTR levels [28], another study suggests that an increase in DTR increases the risk of hospitalization for hypertensive patients, and the cumulative lag effect is more pronounced than a single-day lag [29]. Luo et al. quantified the impact of extreme DTR, identifying it as an independent risk factor for daily mortality. Ultra low DTR had a more direct impact on increasing the population mortality rate compared to ultra high DTR, aligning with some of our findings [30]. A research from East Asia showed that DTR levels were lower in summer and winter [31]. Chen et al. established a significant relationship between seasonal factors and metabolic syndrome, where winter correlated positively with blood pressure and fasting blood glucose, while summer correlated positively with metabolic syndrome, including hyperlipidemia [32]. The risk of diabetes and hyperlipidemia was higher in summer, which may be related to higher lipoprotein lipase activity [33]. As mentioned earlier, the relatively low summer DTR levels in Wuhu City indirectly explain the increased risk of death for metabolic syndrome patients with ultra low DTR. In cold winters, low physical activity and high food intake levels could contribute to weight gain, indirectly raising the risk of MetS death. [34].

The results of seasonal stratification analysis are similar to the overall trend, with ultra low DTR during the cold season increasing the mortality rate of MetS patients. Some studies have pointed out that the minimal difference between the highest and lowest daily temperatures may lead to failure in body temperature regulation, including sweating, vasodilation, and increased heart rate [35], which can affect the circulatory system and lead to significant blood pressure fluctuations, especially for hypertensive patients. This fluctuation may increase the cardiovascular burden and increase the risk of death. Secondly, the immune system may become less active under cold conditions, increasing people’s risk of infection [36], especially leading to complications in patients with high blood sugar [37]. Finally, in winter, the activation of BAT improves cold resistance at the cost of heat resistance. This may trigger anxiety and psychomotor excitement, negatively affecting emotions [38]. This is particularly important for hypertensive patients, as psychological stress can lead to short-term or long-term increases in blood pressure [39]. At the same time, ultra low and low DTR exposure raised the MetS death risk in women, and ultra low DTR was also significantly associated with the danger of MetS death among the elderly. A study from South Korea showed that the elderly’s and women’s death risk was significantly higher than that in other population groups [40]. We tentatively assume that this difference is due to the different adaptability of different populations to temperature changes. Keatinge found that the increase of platelets, red blood cells and viscosity is related to the body’s temperature regulation to adapt to temperature changes [41], while Lim ‘s study pointed out that the ability of the human to effectively control and regulate body temperature decreased as the ages grew [42], which may be the reason why the elderly are more sensitive to DTR.

The association between T mean and MetS mortality risk is shown in Table 3. We found that exposure to extreme T mean (ultra low and ultra high) increases the risk of death from MetS. An all-cause study found that as T mean increases or decreases, the risk of diabetes - related death increases [43]. Secondly, a study from Northeast China showed that increased temperature caused by human activities was significantly associated with the risk of MetS death. The region has a temperate monsoon climate and the subtropical zone is part of the temperate zone, which is consistent with our findings [44]. At the same time, another study showed that long-term exposure to higher levels of temperature would increase the risk of MetS. When the annual temperature increased by 1 ℃, the risk of fasting blood glucose increased by 33% [15]. However, a study from Guangdong, China, reported no statistical correlation between T mean and MetS, but did find significant correlations with blood pressure and fasting blood glucose, with a lag effect [25]. Currently, there is limited research on the connection between temperature and MetS. Reviewing the available data, it becomes evident that insulin resistance is widely regarded as the foundation of MetS pathogenesis. An editorial pointed out that metabolic markers such as triglycerides, high-density lipoprotein cholesterol levels and blood glucose could increase the risk of suffering from MetS [45]. Sergio Valdés found a significant association between ambient temperature and the prevalence of abnormal blood glucose and insulin resistance in Spanish adults [46]. This indirectly suggests that temperature may influence the risk of MetS-related mortality. Additionally, studies have confirmed that changes in temperature can affect sympathetic nervous system activity and plasma renin activity. Low temperatures can also influence oxidative stress and antioxidant defense systems [47]. Li et al. thought that high temperature may result in a highly stressful state of circulatory system, which will lead to increased blood viscosity andtotal peripheral vascular resistance, as well as decreased vascular elasticity. Thus, it finally would increase the risk of MetS death [48].

In the hot season, we discovered an interesting phenomenon. A lower T mean shows a protective effect on MetS patients, while ultra high T mean increases the risk of MetS death. A review has highlighted the role of active physical activity in preventing and treating metabolic syndrome [49]. We noticed that the protective effect of lower T mean is particularly pronounced at around 20 degrees Celsius, which is a comfortable temperature for the human body. At this temperature, people are more inclined to engage in physical activity, indirectly reducing the MetS mortality rate. Our stratification results are shown in Fig. 6. The effects of T mean values on different gender groups are roughly similar, but it is worth noting that women seem to be more sensitive to high temperature than men, and ultra high T mean values have an immediate impact. Research has shown that women typically have a higher body fat percentage than men, making them more adaptable to low temperatures and less prone to sweating in hot environments. This may explain their reduced tolerance to high temperatures [50, 51]. The age-based stratification results indicate that, in comparison to young individuals, the elderly exhibit a delayed response to various T mean levels, underscoring their heightened sensitivity to temperature fluctuations. This aligns with our common understanding, as younger individuals often possess better physical resilience and adaptability to temperature changes. Research has consistently shown that extreme temperatures, both hot and cold, significantly elevate mortality rates among the elderly [52]. This is in line with our own findings. Furthermore, we observed that the elderly demonstrate a delayed response to ultra-high T mean levels on the day of exposure. A study on the environment and elderly people reported that elderly people are more sensitive to immediate exposure to high temperatures, which indirectly explains this phenomenon [53].

In our analysis of RH, we did not find a significant correlation with the risk of MetS-related mortality. However, when we conducted subgroup analysis by dividing the year into hot and cold seasons, we observed that ultra high RH increased the mortality rate of MetS during the hot season. At present, there are few studies on RH and MetS, a randomized controlled experiment found that diabetes patients could not tolerate humid and warm air with humidity more than 50%, and high humidity would increase the blood flow of pat [54]. This is likely because individuals with diabetes have reduced capacity to regulate their body temperature, indirectly increasing their risk in humid environments [55]. In addition, only one study shows that there is a positive correlation between RH and MetS, women living in high RH are more likely to suffer from MetS [56]. But no such association was found in male, young and elderly, which is consistent with some of our findings. The results of the study for the female population are different, which is quite understandable. Quito, with its tropical rainforest climate characterized by high temperatures and abundant year-round precipitation, maintains consistently high relative humidity. Compared to subtropical monsoon humid regions, the average temperature in Quito is higher. Our analysis of the mean temperature (T mean) revealed an increased risk of MetS-related mortality among women at higher T mean levels, possibly contributing to higher mortality rates among women exposed to elevated temperatures, leading to these contrasting findings. Due to the limited existing research on RH and MetS, further investigation is needed to clarify the conflicting results.

This article uses advanced statistical methods to control the influencing factors of atmospheric pollutants, evaluate the relationship between RH, T mean, and DTR with MetS mortality risk, and further stratify the data by gender and age to provide more detailed results. There is relatively little research on the impact of meteorological conditions on MetS, which is a new research direction. However, this study still has some limitations. Firstly, our meteorological data is sourced from meteorological stations rather than personal environmental exposure, potentially leading to exposure misclassification and an underestimation of climate’s impact. Secondly, the types of confounding factors included in this study model are limited. Due to the confidentiality of the data, other potential confounding factors such as the deceased’s occupation, dietary habits, and socio-economic information cannot be obtained, which may limit the biological significance of the results. Furthermore, this study primarily focuses on short-term exposure to meteorological factors and may overlook inconsistent results due to long-term exposure. Except for RH, water vapor pressure may be a better indicator of atmospheric humidity conditions, and in future research, further analysis of the long-term effects of exposure to each meteorological factor is needed. Finally, further research is needed in molecular biology to investigate the causal relationship between MetS mortality and meteorological factors.

This study explores the link between short-term climate changes and MetS mortality, offering public health insights for metabolic syndrome prevention and improved resource allocation during high-risk periods.

## Conclusions

This study revealed that both DTR and Tmean elevate the overall risk of MetS-related mortality in the entire population of Wuhu. Lower DTR has a more pronounced effect on women and the elderly, and ultra low and high T mean is a risk factor for MetS mortality in women and men. Additionally, the elderly need to pay more attention to temperature changes, and different levels of T mean increase the risk of death as well. MetS patients should avoid exposure to high temperature and relative humidity.

### Electronic supplementary material

Below is the link to the electronic supplementary material.

Supplementary Material 1

## Data Availability

Access to any dataset used can be obtained by contacting the corresponding author.
