# Supplementary material for: Research on the relationship between common metabolic syndrome and meteorological factors in Wuhu, a subtropical humid city of China
Source: BMC Public Health. 2023 Nov 29;23:2363. doi: 10.1186/s12889-023-17299-8 (PMC10685562; doi:10.1186/s12889-023-17299-8)

Supplementary Figure 1 The 3D graph, and overall exposure-response association curve between DTR, RH, T mean and metabolic syndrome mortality in the cold season.


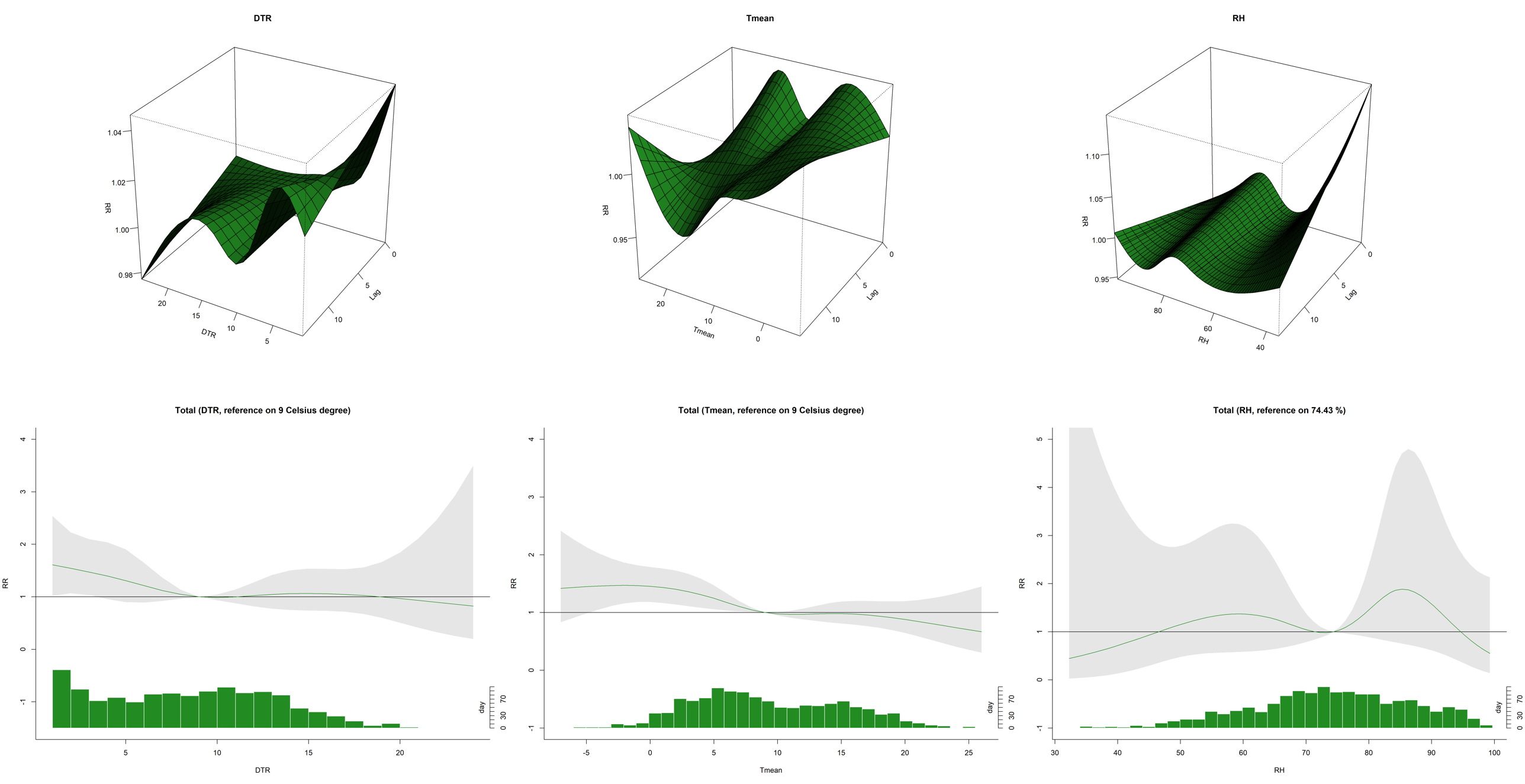


Supplementary Figure 2 The 3D graph, and overall exposure-response association curve between DTR, RH, T mean and metabolic syndrome mortality in the hot season.


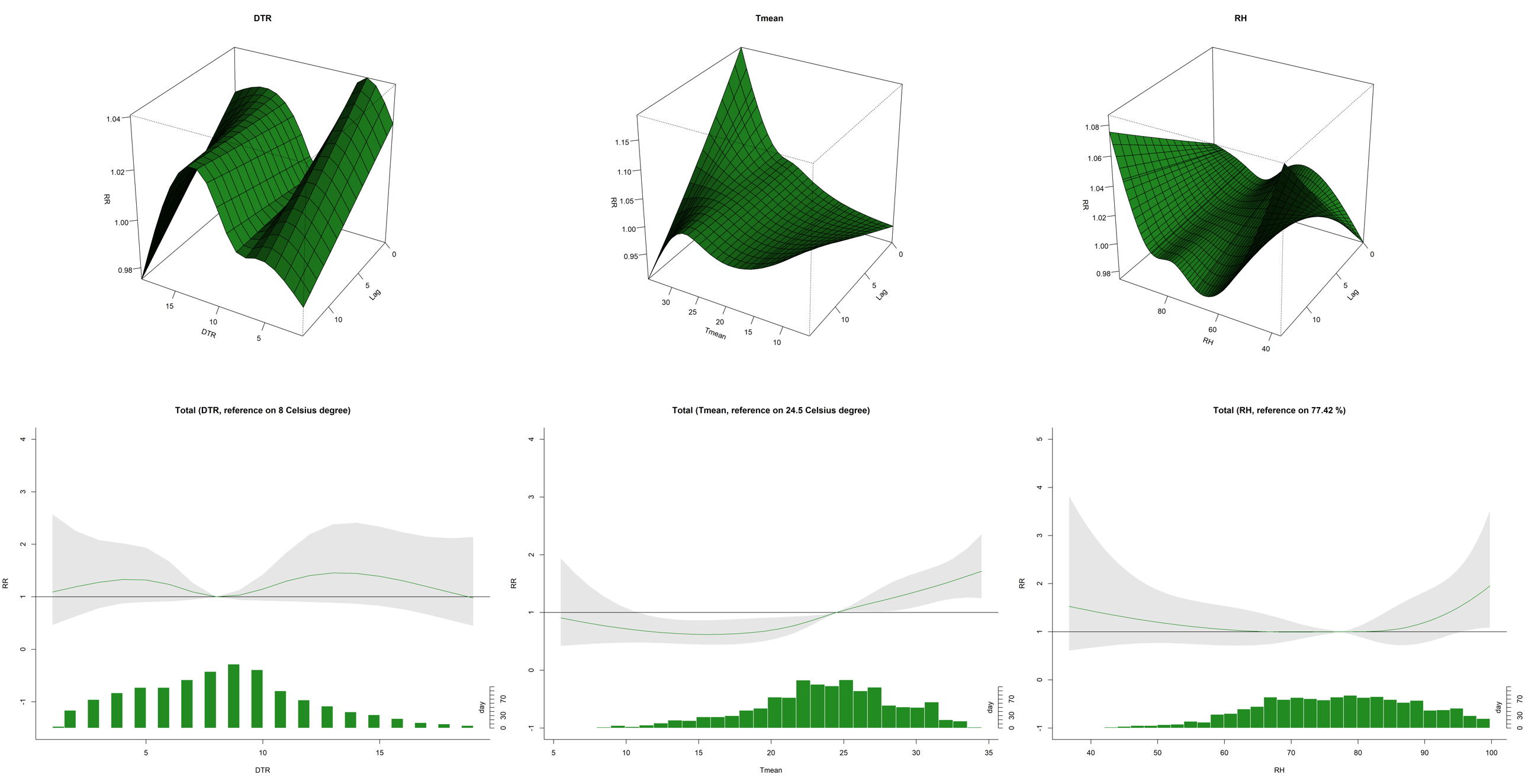

Supplement: Supplementary file 1 — Supplementary Material 1 [file 12889_2023_17299_MOESM1_ESM.docx]
